# TWIST1 bridges p53 and MDM2 and impairs the efficacy of MDM2 inhibitors

**DOI:** 10.1186/s13046-026-03780-w

**Published:** 2026-07-16

**Authors:** Sara Piccinin, Bernadetta Szaboova, Flavia Pivetta, Elisa Del Savio, Elena Belli, Simona Gloazzo, Elena Doriguzzi Breatta, Davide Baldazzi, Camillo Rosano, Luca Sigalotti, Roberta Maestro

**Affiliations:** 1 Unit of Oncogenetics and Functional Oncogenomics, Centro di Riferimento Oncologico di Aviano IRCCS (CRO Aviano), Aviano, Italy; 2 Proteomics and Mass Spectrometry Unit, Ospedale Policlinico San Martino, Genova, Italy

**Keywords:** MDM2 inhibitors, TWIST1, Sarcoma, p53, MDM2 amplification

## Abstract

**Background:**

Tumors that retain wild-type *TP53* and rely on MDM2 overexpression to dampen the p53 response were considered prime candidates for therapies based on MDM2 inhibitors (MDM2i). However, clinical trials to date have been disappointing, with limited improvements in progression-free survival, pointing to the existence of intrinsic resistance mechanisms. Building on our previous work demonstrating that TWIST1 attenuates the p53 response in sarcomas, we hypothesized that TWIST1 plays a role in modulating MDM2i efficacy.

**Methods:**

RNA-sequencing data were integrated with cytotoxicity measurements across a large panel of *TP53* wild-type sarcoma cell models. Perturbation experiments were performed using shRNA or CRISPR/Cas9-mediated inhibition, as well as ectopic TWIST1 expression. Cell viability, pathway activation, and transcriptional profile following MDM2i treatment were evaluated in TWIST1-proficient and -deficient models. Co-precipitations, TurboID, molecular docking experiments, and in vitro and in vivo functional assays were employed to characterize the interplay between TWIST1, p53, and MDM2.

**Results:**

*TWIST1* expression correlated with reduced sensitivity to multiple MDM2i. TWIST1 inhibition enhanced p53 pathway activation and cell death in response to MDM2i, while TWIST1 overexpression conferred resistance. Mechanistically, we found that TWIST1 directly binds both p53 and MDM2, forming a trimeric complex that facilitates p53:MDM2 interaction, thereby promoting p53 degradation and limiting MDM2i efficacy. Notably, harmine, a compound that promotes TWIST1 degradation, phenocopied the effects of TWIST1 genetic inhibition in augmenting MDM2i sensitivity.

**Conclusion:**

TWIST1 confers resistance to MDM2i in *TP53* wild-type sarcomas. Targeting TWIST1 restores p53 pathway activation and sensitizes sarcoma cells to MDM2 blockade, establishing TWIST1 as a promising predictive biomarker and therapeutic vulnerability for improving MDM2i efficacy.

**Supplementary Information:**

The online version contains supplementary material available at https://doi.org/10.1186/s13046-026-03780-w.

## Background

The p53 tumor suppressor pathway is a central guardian of genomic integrity, activated in response to a range of cellular stresses, including oncogene activation. Inactivation of this pathway represents a major roadblock that must be overcome during cellular transformation. While the majority of human cancers achieve this through inactivating mutations in the *TP53* gene, a subset of tumors including most sarcomas retain *TP53* wild-type and instead rely on alternative mechanisms to dampen p53 activity. Among these, MDM2 overexpression is the most prevalent strategy. MDM2 acts as a potent negative regulator of p53 by inhibiting its transcriptional activity and facilitating its ubiquitination and subsequent proteasomal degradation [1–3].

*MDM2* amplification and overexpression is commonly observed in sarcomas and it is a hallmark of well-differentiated (WDLPS) and dedifferentiated liposarcomas (DDLPS), intimal sarcomas (IS), and low-grade osteosarcomas [4–7]. The relevance of MDM2 in attenuating the p53 response in *TP53* wild-type tumors has led to the development of MDM2 small molecule inhibitors (MDM2i). These compounds, by interfering with the p53:MDM2 interaction, relieve p53 from MDM2-mediated inhibition and were expected to trigger massive apoptosis, cell cycle arrest, and senescence in *TP53* wild-type tumors [8–12]. Tumors significantly relying on MDM2-mediated inhibition of p53, such as WDLPS, DDLPS and IS, were considered the prime candidates for this type of treatment. Unfortunately, despite very promising preclinical evidence, MDM2i have not fully met the initial great expectations in clinical trials [13, 14]. In fact, although important objective responses were observed in some patients, MDM2i have thus far failed to show improved progression-free survival over standard of care [14–17]. The underlying biological reasons for the failure of these trials to meet their primary endpoint, despite evidence of notable clinical benefit in some patients, remain unclear. Proposed resistance mechanisms include: the emergence of secondary p53-inactivating mutations under therapeutic pressure; intratumoral heterogeneity in MDM2 amplification; activation of the p53-MDM2 negative feedback loop, whereby p53 stabilization induces transcriptional upregulation of MDM2, which in turn can sequester and titrate out the inhibitor; and differential expression of p53 regulatory proteins, including MDM4 (MDMX), which inhibits p53 transcriptional activity and, in complex with MDM2, enhances p53 degradation [18–21]. New approaches based on degraders/PROTACS are emerging [22](e.g., trial NCT05775406), but in vitro data indicate that, despite the potential eligibility (*TP53* wild-type and MDM2 expression), not all cell lines are induced to death by these degraders [23], highlighting the presence of undefined primary resistance mechanisms.

We have previously shown that TWIST1 plays a critical role in antagonizing the p53 response in sarcomas. TWIST1 is a transcription factor involved in embryonic mesoderm development [24]. Although typically silent in adult tissues, TWIST1 is frequently reactivated in tumors, where it promotes cellular plasticity and enables bypass of failsafe mechanisms through inhibition of the p53 and RB tumor suppressor pathways [25–28]. In particular, TWIST1 is overexpressed in a significant fraction of sarcomas, where it attenuates the p53 response by interfering with both the oncogenic induction of p14ARF and key p53 phosphorylations that regulate its stability [26]. In this work, we further demonstrate that TWIST1 reduces the efficacy of MDM2i in *TP53* wild-type, *MDM2*-amplified and non-amplified sarcoma models. Mechanistically, TWIST1 directly binds both p53 and MDM2, forming a trimeric complex that facilitates MDM2-mediated degradation of p53. These findings identify TWIST1 as a critical modulator of MDM2 inhibitor response and highlight its potential as a predictive biomarker and therapeutic vulnerability.

## Methods

### Cell cultures

*TP53* wild-type sarcoma cell lines utilized in this study include: HT1080, VA-ES-BJ, U2OS, G401, A204, SW872, HEMCSS, CADOES (*MDM2* non-amplified cell lines); SJSA-1, LPS141, PIS-1, 93T449, 94T778, LIPO573, LPS246, LPS863, LIPO815, and LPS224 (*MDM2*-amplified cell lines). HT1080, VA-ES-BJ, U2OS, G401, A204, SW872, SJSA-1, 93T449, and 94T778 were from the American Type Culture Collection (ATCC, Manassas, VA, USA). HEMCSS were obtained from the RIKEN BioResource Research Center (Tsukuba, Japan) and CADOES were from DSMZ (Braunschweig, Germany). LPS141 were kindly provided by J.A. Fletcher (Harvard Medical School, Boston, USA). PIS-1 were from the Japanese Collection of Research Bioresources Cell Bank (Ibaraki, Osaka, Japan). LIPO573, LPS246, LPS863, LIPO815, and LPS224 were from the MD Anderson Cancer Center cell line repository (Huston, TX, USA). H1299 and HEK293T were from ATCC. The p53-null HCT116 were kindly provided by B. Vogelstein (Johns Hopkins University, Baltimore, USA). *TP53* and *MDM2* gene status was verified by whole exome sequencing for the in-house cell panel and by interrogation of COSMIC v102 (https://cancer.sanger.ac.uk/cosmic/login) and the *TP53* database (https://tp53.cancer.gov/) for the Genomics of Drug Sensitivity in Cancer (GDSC, https://www.cancerrxgene.org/) cell line cohort. All cell lines were maintained in a humidified incubator with 5% CO2. Cell lines were grown in DMEM medium except for 94T778, 93T449, PIS-1, and SJSA-1, which were grown in RPMI medium. Culture media were supplemented with 10% heat-inactivated fetal bovine serum (FBS) and 8 µg/ml gentamycin. All cell lines were periodically STR-authenticated and confirmed to be free of mycoplasma contamination.

### RNA-sequencing and gene set enrichment analysis

RNA was extracted with miRNeasy Mini Kit (Qiagen, Hilden, Germany) and RNA-sequencing was performed essentially as in Sigalotti et al. [29]. Briefly, 250 ng total RNA were used for library preparation with the Illumina Stranded Total RNA Prep with Ribo-Zero Plus (Illumina, San Diego, CA, USA) following manufacturer’s instructions. Sequencing was performed on an Illumina NextSeq550 apparatus to an average of 70 million paired-end reads per sample. The quality of the row sequencing data was evaluated with the FastQC software (RRID: SCR_014583). Reads were aligned to the hg38 reference genome (GRCh38.p13) with STAR v2.7.3.a (RRID: SCR_004463) [30]. RSEM v1.3.1 (RRID: SCR_000262) [31] was utilized to quantify the reference transcripts and a custom script was utilized to calculate pTPM (protein-coding transcripts per million). pTPM data for the GDSC dataset were retrieved from the DepMap Public 24Q4 dataset (Broad 2024) [32]. Differential expression analysis was computed as in Sigalotti et al. [29] using the DESeq2 v1.32.0 package (RRID: SCR_015687) [33] incorporated into DElite [34]. To compare cell lines with different sensitivities to MDM2i, differential expression analysis was conducted between cell lines in the upper and lower IC50 tertiles. Pre-ranked gene set enrichment analysis (GSEA) [35] was carried out with the FGSEA v1.18.0 package (RRID: SCR_020938) using 10,000 permutations. Gene ranking was based on the composite score –log10(p-value) × sign(log2FC) to incorporate both statistical significance and directionality of expression change as previously described [29]. Gene sets used in this work included genes upregulated or downregulated by ectopic *TWIST1* expression (|log2FC|≥1, padj ≤ 0.01) [36], the p53 pathway signature of the MSigDB hallmark gene set collection [37] and a signature of genes positively regulated by p53 [38].

### Inhibitors and cell viability assay

The following MDM2 small molecule inhibitors (MDM2i) were used in this study: nutlin-3a (Selleckchem, Houston, TX, USA), DS-3032 (Chemietec, Bergenfield, IN, USA), SAR405838, RG7388, and BI 907828 (MedChemExpress, Monmouth, NJ, USA). Harmine was from Thermo Fisher Scientific (Thermo Fisher Scientific, Waltham, Massachusetts, US). TAK-243, MLN4924, and MG132 (MedChemExpress) were used to inhibit global ubiquitination, neddylation, and proteasome-mediated degradation, respectively. All compounds were dissolved in DMSO (Sigma-Aldrich, St. Louis, Missouri, US) at 100X or 1000X stock concentration. For cytotoxicity assays, cells were seeded at a density of 3.5 × 10^3^ cells/well in 96-well plates. The following day, cells were treated in quadruplicate or sextuplicate with MDM2i. For the calculation of IC50, cells were treated with MDM2i at two-fold serial dilutions. Seventy-two hours later, cells were fixed with trichloroacetic acid and processed for sulforhodamine B (SRB) (Sigma-Aldrich) cell viability assay according to standard protocol [39] with minor modifications; absorbance at 550 nm was read using a Spark Multimode Microplate Reader (TECAN Group LTD, Mannedorf, CH). GraphPad was used to calculate the IC50. IC50 values for nutlin-3a of the GDSC sarcoma cell line cohort (GDSC1 dataset) were retrieved from the Genomics of Drug Sensitivity in Cancer database (https://www.cancerrxgene.org/compound/Nutlin-3a%20(-)/1047/overview/ic50?screening_set=GDSC1). For the cytotoxicity assay under 3D culture conditions, cells were first treated for 24 h with MDM2i (100 nM DS-3032; 500 nM RG7388), then plated in semisolid medium as previously described [26]. Colony growth was assessed three weeks after plating by counting colonies with a diameter > 100 μm.

### Plasmids and cell line engineering

A pre-validated *TWIST1*-specific shRNA and a shRNA targeting the Luciferase irrelevant sequence [26, 27, 40] were used to knockdown *TWIST1* expression and as a control, respectively. shRNAs were cloned in the pLVTHM lentiviral vector. Viral infections were as previously described [26, 41]. CRISPR/Cas9-mediated *TWIST1* knockout was obtained by using two RNA guides targeting the 5’end of *TWIST1* DNA (guide # 1: 5′-CTGTCGTCGGCCGGCGAGAC-3'; guide #2: 5′-TTGCTCAGGCTGTCGTCGGC-3′). A guide targeting the irrelevant Luciferase gene (5′-AATGGGGCTATACCCGCTAC-3′) was used as a control. Guides were designed using the CRISPick tool (https://www.zlab.bio/guide-design-resources) and cloned into BbsI-digested pSpCas9(BB)-2A-GFP vector (a gift from Feng Zhang; Addgene plasmid # 48138) [42]. Plasmids were transfected with the Lipofectamine 3000 transfection reagent (Thermo Fisher Scientific) and, 48 h post-transfection, GFP-positive cells were FACS-sorted using a BD FACSAria (BD Bioscience, Franklin Lakes, New Jersey, US) and single-cell cloned. Knocked out clones were screened by western blotting and characterized by DNA sequencing. To map TWIST1 interaction domains we used a mouse *Twist1* construct. Human and mouse TWIST1 proteins shows 100% identity in the C-terminal region encompassing the bHLH and the WR domains. Mouse *Twist1* full-length cDNA and relative deletion mutants (ΔbHLH corresponding to Δ103–169; ΔWR corresponding to Δ175–194) were cloned in the pLPC-5xFLAG retroviral vector and in the pCS2-6xMYC plasmid. The region corresponding to the Twist1 WR domain (amino acids 175–199) was cloned in-frame into the thioredoxin active-site loop to enhance domain exposure, as previously described [26]. Human *TWIST1* cDNA and GFP were cloned into the FLAG-TurboID vector (a gift from Feng-Qian Li & Ken-Ichi Takemaru; Addgene plasmid # 124646). pCMV-human *MDM2* was a gift from Bert Vogelstein (Addgene plasmid # 16441) [43]. *MDM2* was cloned into the pCS3-6xMYC vector. MDM2 deletion mutants were generated by site-directed mutagenesis (QuikChange ^TM^ Site Directed Mutagenesis Kit). Human *TP53* wild-type and *TP53*ΔMDM2 were cloned in pLPC-5xFLAG. The *TP53*ΔMDM2 allele, carrying the double L22Q/W23S mutation that impair the N-terminal interaction of p53 with MDM2 [44], was generated by site-directed mutagenesis. GFP, RFP, human *TP53*, *MDM2*, and *TWIST1* (full length and mutants) were cloned into pPSG105 to generate STREP-tag recombinant proteins. STREP-tag cDNAs were subcloned into pCS3 and pLPC for expression in mammalian cells. pGEX-KG *TWIST1*, *MDM2*, *TP53*, and empty vector were used to generate GST-tagged recombinant proteins.

### Antibodies

The antibodies used in this study were: mouse monoclonal anti-p53 (DO1, SCBT Santa Cruz Biotechnology, Texas, TX, USA); rabbit polyclonal anti-p53 (FL393, SCBT); mouse monoclonal anti-TWIST1 (TWIST2C1a, SCBT); rabbit polyclonal anti-MYC (ab9106, Abcam, Cambridge, MA, USA); mouse monoclonal anti-MYC (9E10, Calbiochem, San Diego, CA, USA); mouse monoclonal anti-MDM2 (2A10, Oncogene science, Uniondale, NY, USA); mouse monoclonal anti-MDM2 (SMP14, SCBT); rabbit polyclonal anti-p21 (C-19, SCBT); rabbit monoclonal anti Bax (D2E11, Cell Signaling Technology, Danvers, MA, USA); rabbit polyclonal anti-cleaved Caspase-3 D175 (Cell Signaling Technology); mouse monoclonal anti-STREP-tag (IBA); mouse monoclonal anti-FLAG (M2, Sigma-Aldrich); Streptavidin, horseradish peroxidase (HRP) conjugate (S911, Thermo Fisher Scientific). Monoclonal antibodies targeting γ-Tubulin (GTU-88, Sigma-Aldrich), α-Tubulin (B-5-1-2, Sigma-Aldrich), GAPDH (6C5, SCBT) or Vinculin (42H89L44, Thermo Fisher Scientific) were used for protein loading normalization. Goat anti-rabbit IgG and anti-mouse IgG HRP-labeled (Revvity, Waltham MA, USA) were used as secondary antibodies. For immunofluorescence analysis, anti-p53 (FL393, SCBT), anti-MDM2 (2A10, Oncogene Science), anti-MYC (9E10, Calbiochem), and anti-STREP (StrepMAB-Immo Chromeo 546 Conjugate, IBA) monoclonal antibodies were used to detect p53, MDM2, MYC-tag or STREP-tag TWIST1, respectively. Anti-rabbit (Dylight 633-conjugated, Invitrogen, Carlsbad, CA, USA) and anti-mouse (Alexa Fluor 488- or 594-conjugated, Thermo Fisher Scientific) were used as secondary antibodies.

### Western blot, pull-down assays, and immunofluorescence

Whole cell lysates prepared in RIPA buffer (SCBT) supplemented with protease and phosphatase inhibitors were used for western blot analyses. Nuclear extracts were prepared according to Dignam and Roeder’s protocol [45] with minor modifications. IP lysis buffers (120 or 200mM NaCl, 20mM Tris-HCl pH8, 1 mM EDTA, 0.5% IGEPAL CA-630) supplemented with protease and phosphatase inhibitors was used for co-immunoprecipitation and co-affinity purification experiments. For two-step co-precipitation assays (co-affinity precipitation followed by co-immunoprecipitation), 10-cm cell plates were transfected with MYC-tagged *MDM2* (pCS3-6xMYC, 12 µg) and STREP-tagged *TWIST1* or *GFP* (pCS3-STREP, 3 µg). Forty hours post-transfection, cells were lysed in two-step lysis buffer (100mM NaCl, 20mM Tris-HCl pH7.5, 1% IGEPAL CA-630, plus protease and phosphatase inhibitors), passed several times through a 22-gauge needle, and centrifuged. The supernatant then was incubated overnight with STREP-Tactin sepharose resin (IBA Lifesciences, Göttingen, Germany) at 4 °C. Beads were then washed several times with wash buffer (150mM NaCl, 20mM Tris-HCl pH 7.5, 1% IGEPAL CA-630, plus protease and phosphatase inhibitors).The proteins bound to the beads were eluted by incubation for 2 h at 4 °C in elution buffer (250mM NaCl, 20mM Tris-HCl pH 7.5, 1% IGEPAL CA-630, 2.5 mM desthiobiotin, protease and phosphatase inhibitors). Eluates were then divided in 2 aliquots and subjected to the second round of precipitation using anti MYC-tag or control rabbit IgG antibodies (4 °C, overnight). The following day, protein complexes were recovered with protein A-sepharose (Cytiva Italy srl, Milan, Italy). GST pull-downs with in vitro translated and recombinant proteins were performed essentially as previously described [26]. To assess the ability of recombinant TWIST1 to enhance the interaction between MDM2 and p53 in vitro, GST pull-downs were performed using STREP-tagged MDM2 (25 ng), which was pre-incubated for 2 h at 4 °C with DS-3032 (5 µM) or vehicle control (DMSO) in a binding buffer containing 150 mM NaCl, 20 mM Tris-HCl (pH 7.5), 0.1% IGEPAL CA-630, 1 mM EDTA, and 1 mM DTT, supplemented with protease and phosphatase inhibitors. GST-p53 (0.1 µg) was subsequently added together with increasing amounts (0.1, 0.2 µg) of either STREP-tagged TWIST1 (full-length), STREP/MYC tagged TWIST1ΔWR, or an unrelated STREP-tagged protein (RFP) used as a negative control (STREP-CTRL). Following an additional 2 h incubation at 4 °C, protein complexes were isolated by GST pull-down using Glutathione Superflow Agarose beads (Thermo Fisher Scientific). Proteins were resolved by SDS-PAGE and transferred onto Protran nitrocellulose membrane (Sigma-Aldrich). Western blots were detected by chemiluminescence (LiteUp kit, Euroclone, Pero, Milan, Italy), captured with a ChemiDoc MP apparatus (Bio-Rad, Hercules, CA, USA), and analyzed with the ImageLab software (Bio-Rad). Immunofluorescence was performed essentially as previously described [41]. Images were acquired with a Leica TCS SP8 Confocal system (Leica Microsystems Heidelberg, Mannheim, Germany) and analyzed with the Leica Confocal Software (Leica Application Suite X version 3.1.5.16308).

### Proximity-dependent biotinylation assay (TurboID)

Proximity-dependent biotinylation assays were performed as previously described [46] with minor modifications. Briefly, cells were transfected with FLAG-TurboID constructs. Twenty-four hours post-transfection, 0.5 mM biotin (B4501, Sigma-Aldrich) was added to the culture while control plates received an equal volume of vehicle (DMSO). After 10 min, the biotinylation reaction was stopped by placing the dishes on ice. Cells were washed several times with ice-cold PBS and lysed in lysis buffer (100mM NaCl, 20mM Tris-HCl pH7.5, 1% IGEPAL CA-630). Biotinylated proteins were purified using STREP-Tactin sepharose resin (IBA Lifesciences) and separated by SDS-PAGE.

### In vivo xenograft tumor experiments

Athymic 6-week-old female nude mice (Hsd: Athymic Nude-Foxn1^nu^, Envigo RMS SRL, Udine, Italy) were subcutaneously injected with 2.5 × 10^6^ SJSA-1 or HT1080 cells, either TWIST1-proficient (CTRL) or -deficient (TWIST1 KD). Mice were randomly assigned to receive the MDM2i or vehicle control when tumors reached approximately 100–200 mm³ in volume (since *TWIST1* silencing prolongs tumor latency [26], tumors generated from *TWIST1*-silenced cells reached the target volume later than control cells). Thirteen TWIST1-proficient and -deficient SJSA-1 xenografts were treated with vehicle, and 14 with DS-3032. Twelve TWIST1 KD HT1080 xenografts were treated with vehicle, 12 with DS-3032, and 11 with BI 907828; 8 HT1080 CTRL xenografts were treated with vehicle, 10 with DS-3032, and 13 with BI 907828. Drugs and vehicle were prepared according to the manufacturer’s instructions (MedChemExpress) and administered by oral gavage once daily for 8 consecutive days (DS-3032, 20 mg/kg; BI 907828, 1 mg/kg body weight), followed by treatment discontinuation. Tumor size was measured every 2 days using a caliper, and tumor volume was calculated using the formula: length × width² × 0.5. Growth curves were normalized to baseline tumor volume at treatment initiation (T0) using the ratio Tn/T0, where Tn represents the tumor volume at each time point. Mice were euthanized by CO₂ inhalation at the experimental endpoint. The experimental procedures involving animals were compliant with the 3Rs principles and the European Commission Directive 2010/63/EU, and were approved by the Institutional Animal Care and Use Ethics Committee of CRO Aviano and by the Italian Ministry of Health (#642/2020-PR; #747/2023-PR). The maximal tumor size permitted by the Ethics Committee was 1.2 cm in diameter; this limit was not exceeded.

### Protein modeling and docking simulation

Protein modeling and docking simulations were essentially as previously described [26]. Briefly, three-dimensional structure models of human TWIST1, MDM2, and p53 were built by using the SAM-T08 protein structure prediction server and the Crystallography and NMR System (CNS) suite. Clus Pro 2.0 was used for computational docking of protein structures.

### Statistical analysis

Statistical analyses and IC50 calculations were performed using GraphPad Prism for Windows version 9.3.1 (GraphPad Software, San Diego, CA, USA). An unpaired two-tailed t-test was used to compare the means of two independent groups. Where appropriate, a logarithmic transformation was applied prior to statistical analysis to normalize the data distribution. Correlation analyses were calculated using Spearman’s rank correlation test, with exact p-value estimation. One-way ANOVA test was used to assess differences among the means of multiple independent groups. Tumor growth curves were analyzed using a two-way repeated-measures ANOVA. A threshold of 0.05 was used to determine statistical significance. All results were confirmed in at least three independent experiments.

### Data availability

Transcriptome data generated in this study are provided as Supplementary Table ST2. Raw data are available upon request from the corresponding author. For the GDSC sarcoma cell line cohort, pTPM were retrieved from the DepMap Public 24Q4 dataset (https://depmap.org/portal/data_page/?tab=allData&releasename=DepMap%20Public%2024Q4&filename=OmicsExpressionProteinCodingGenesTPMLogp1.csv) and IC50 values for nutlin-3a (GDSC1 dataset) retrieved from the GDSC database (https://www.cancerrxgene.org/compound/Nutlin-3a%20(-)/1047/overview/ic50?screening_set=GDSC1).

## Results

### *TWIST1* expression correlates with reduced sensitivity to MDM2i in *TP53* wild-type sarcoma cells

**Fig. 1:**
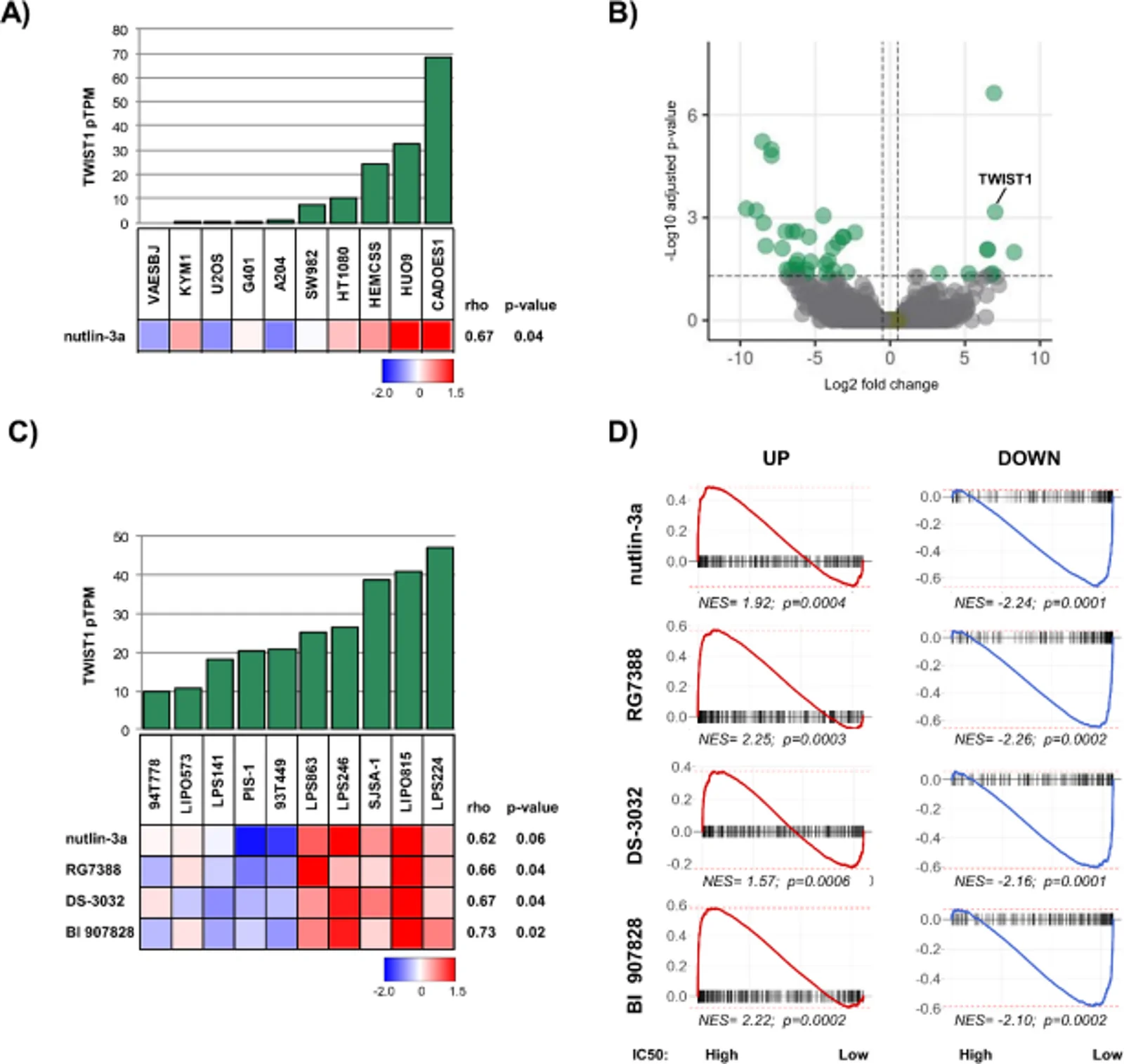
*TWIST1*expression correlates with reduced sensitivity to MDM2i in*TP53*wild-type sarcoma cells. **A** Correlation between *TWIST1* expression (pTPM, bar plot) and nutlin-3a IC50 values (heatmap reporting z-scored IC50 values) in the *TP53* wild-type/*MDM2* non-amplified sarcoma cell lines of the GDSC cell line collection. **B** Volcano plot showing that *TWIST1* is among the top overexpressed genes in GDSC cell lines that responded poorly to nutlin-3a. Differential expression analysis was performed by comparing cell lines in the upper and lower tertiles of IC50 values. **C** Correlation between *TWIST1* (pTPM) and the IC50 values for the indicated MDM2i across our cohort of *TP53* wild-type/*MDM2*-amplified sarcoma cell lines. **D** Pre-ranked GSEA analyses showing that *TWIST1*-associated gene signatures (UP, genes upregulated or DOWN, genes downregulated upon TWIST1 overexpression) were enriched in the cell lines exhibiting reduced sensitivity (higher IC50) to the indicated MDM2i

Building on previous findings that TWIST1 is overexpressed in soft tissue sarcomas and dampens the p53 response, we hypothesized that TWIST1 expression influences the sensitivity of sarcoma cells to MDM2i. To address this hypothesis, we first interrogated the Genomics of Drug Sensitivity in Cancer (GDSC) database, retrieving nutilin-3a sensitivity (IC50) and *TWIST1* gene expression levels (pTPM, protein-coding transcripts per million) across *TP53* wild-type/*MDM2* non-amplified sarcoma cell lines exhibiting variable expression of the nutlin-3a target, MDM2 (Supplementary Figure S1). Intriguingly, a correlation between *TWIST1* expression and reduced nutlin-3a response (higher IC50) was observed (Fig. 1A, Supplementary Table ST1). Indeed, *TWIST1* was among the top overexpressed genes in least sensitive cells (Fig. 1B, Supplementary Table ST2A).

We then probed a well-characterized in-house collection of *TP53* wild-type/*MDM2*-amplified sarcoma cell lines that includes cell models of well-differentiated and dedifferentiated liposarcoma (93T449, 94T778, LPS141, LPS246, LPS863, LPS224, LIPO573, LIPO815), intimal sarcoma (PIS-1), and osteosarcoma (SJSA-1) (Supplementary Figure S1). Cell lines were transcriptionally profiled by RNA sequencing to quantify *TWIST1* gene expression levels (pTPM) and evaluated for MDM2i sensitivity by measuring the IC50 for nutlin-3a, RG7388 (idasanutlin), DS-3032 (milademetan), and BI 907828 (brigimadlin). The trend of correlation between *TWIST1* expression and reduced sensitivity to MDM2i was confirmed also in this setting (Fig. 1C), supporting an inverse association between *TWIST1* and MDM2i efficacy, regardless of MDM2 amplification status.

Moreover, for each MDM2i, we compared the least and the most sensitive cell models (upper vs. lower IC50 tertile) and performed pre-ranked Gene Set Enrichment Analysis (GSEA) on differentially expressed genes using TWIST1-specific gene signatures [36]. In the least sensitive group of cell lines, signatures composed of genes positively (UP) or negatively (DOWN) regulated upon ectopic *TWIST1* expression were enriched or depleted, respectively (Fig. 1D, Supplementary Table ST2B). These results are consistent with the potential role of TWIST1 in tuning MDM2i activity in sarcoma cells.

### TWIST1 affects MDM2i activity

**Fig. 2:**
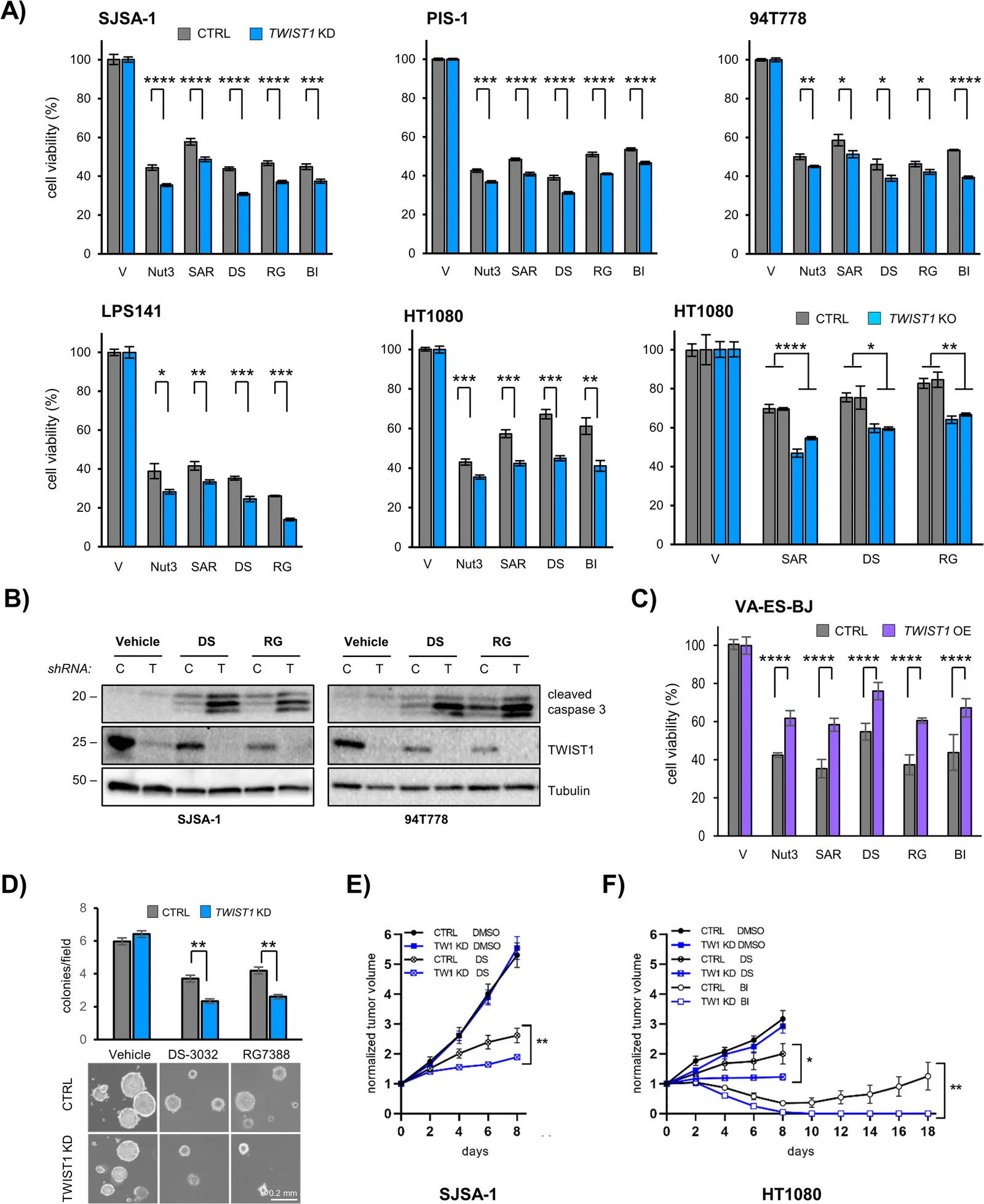
TWIST1 inhibition sensitizes *TP53 *wild-type sarcoma cells to MDM2i. **A ***TP53* wild-type sarcoma cells, including *MDM2*-amplified and non-amplified (HT1080) cell lines, were depleted for *TWIST1* expression by either using shRNA-mediated gene silencing (*TWIST1* KD) or CRISPR/Cas9 knockout (*TWIST1* KO) and then challenged with the indicated MDM2i or vehicle (V, DMSO). Cell viability was measured 48 h post-treatment. Representative dose-response curves are provided in Supplementary Figure S2. **B ***TWIST1* depletion enhances the apoptotic response to MDM2i. *TWIST1*-silenced (T) or control (C) cells were treated with the indicated MDM2i for 24 h. Apoptosis was evaluated by immunoblotting for cleaved caspase-3. Tubulin served as a loading control. **C** Ectopic expression of *TWIST1* in VA-ES-BJ cells confers resistance to MDM2i. Cell viability after 48 h treatment is shown. **D ***TWIST1* knockdown reduces cell viability under MDM2i pressure in 3D culture. TWIST1-proficient and -deficient SJSA-1 cells were treated for 24 h with DS-3032, RG7388, or vehicle (V, DMSO) before plating in semisolid medium. The plot shows the mean number of colonies per field (± SEM) at day 20 post-plating (colony size cutoff: >100 μm). Representative colony fields are shown. **E**,** F** TWIST1 affects sensitivity to MDM2i in vivo. Immunocompromised mice were xenografted with *TWIST1*-silenced (TW1 KD) or control (CTRL) SJSA-1 (E) or HT1080 cells (F). When tumors reached the target size (approximately 100–200 mm³), mice were randomized to receive DS-3032 (20 mg/kg), BI 907828 (1 mg/kg), or vehicle, as indicated. Complete and prolonged tumor regression following the 8-day treatment with BI 907828 was observed in *TWIST1*-silenced HT1080 xenografts, whereas 9 of 13 control xenografts resumed growth within a few days after treatment cessation. Data are presented as mean tumor volume normalized to the volume at day zero of treatment ± SEM. Abbreviations. Nut3, nutlin-3a; SAR, SAR405838; DS, DS-3032; RG, RG7388; BI, BI 907828. Drug concentrations used in this figure are provided in Supplementary Table ST3. All results were confirmed in at least three independent biological replicates. Statistical significance is indicated as follows: *, *p* ≤ 0.05, **, *p* ≤ 0.01, ***, *p* ≤ 0.001, ****, *p* ≤ 0.0001

To delve deeper into this correlation, we inhibited endogenous *TWIST1* expression in *TP53* wild-type sarcoma cell models, including both *MDM2*-amplified and non-amplified cell lines. TWIST1 depletion, by either gene knock-down (KD) using a validated shRNA [26, 27, 40] or by CRISPR/Cas9-mediated gene knock-out (KO), led to increased sensitivity to MDM2i, as evidenced by reduced cell viability and enhanced Caspase-3 activation (Fig. 2A-B, Supplementary Figure S2, Supplementary Table ST3). Conversely, ectopic TWIST1 expression in VA-ES-BJ, a *TP53* wild-type sarcoma cell line in which TWIST1 is expressed at negligible levels, reduced the efficacy of MDM2i (Fig. 2C).

The enhanced sensitivity to MDM2i observed upon TWIST1 depletion was confirmed also under 3D culture conditions. In fact, in the presence of MDM2i, *TWIST1*-silenced SJSA-1 cells formed fewer and smaller colonies compared to TWIST1-proficient cells (Fig. 2D). Moreover, xenograft experiments showed that *TWIST1*-silenced SJSA-1 and HT1080 cells were more sensitive to MDM2i than control cells, indicating that TWIST1 also modulates the response to MDM2 inhibition in vivo (Fig. 2E-F, Supplementary Table ST4).

Notably, long-term post-treatment monitoring in the HT1080 model showed that, following an 8-day treatment with BI 907828, 9 of 13 xenografts derived from control cells resumed growth within a few days after treatment cessation, whereas none of the 11 xenografts derived from *TWIST1-*silenced cells exhibited tumor regrowth. These findings suggest that TWIST1 depletion is associated with a more durable tumor response upon treatment discontinuation (Fig. 2F).

Interestingly, we observed that harmine, a pleiotropic natural alkaloid proposed as a TWIST1 inhibitor due to its ability to promote TWIST1 protein degradation [47, 48], phenocopies the effects of *TWIST1* gene silencing in terms of modulation of MDM2i sensitivity (Supplementary Figure S3). This observation adds support to the notion that TWIST1 inhibition, whether achieved genetically or chemically, enhances the cytotoxic activity of MDM2i.

### TWIST1 impinges upon p53:MDM2 binding and p53 ubiquitination

**Fig. 3:**
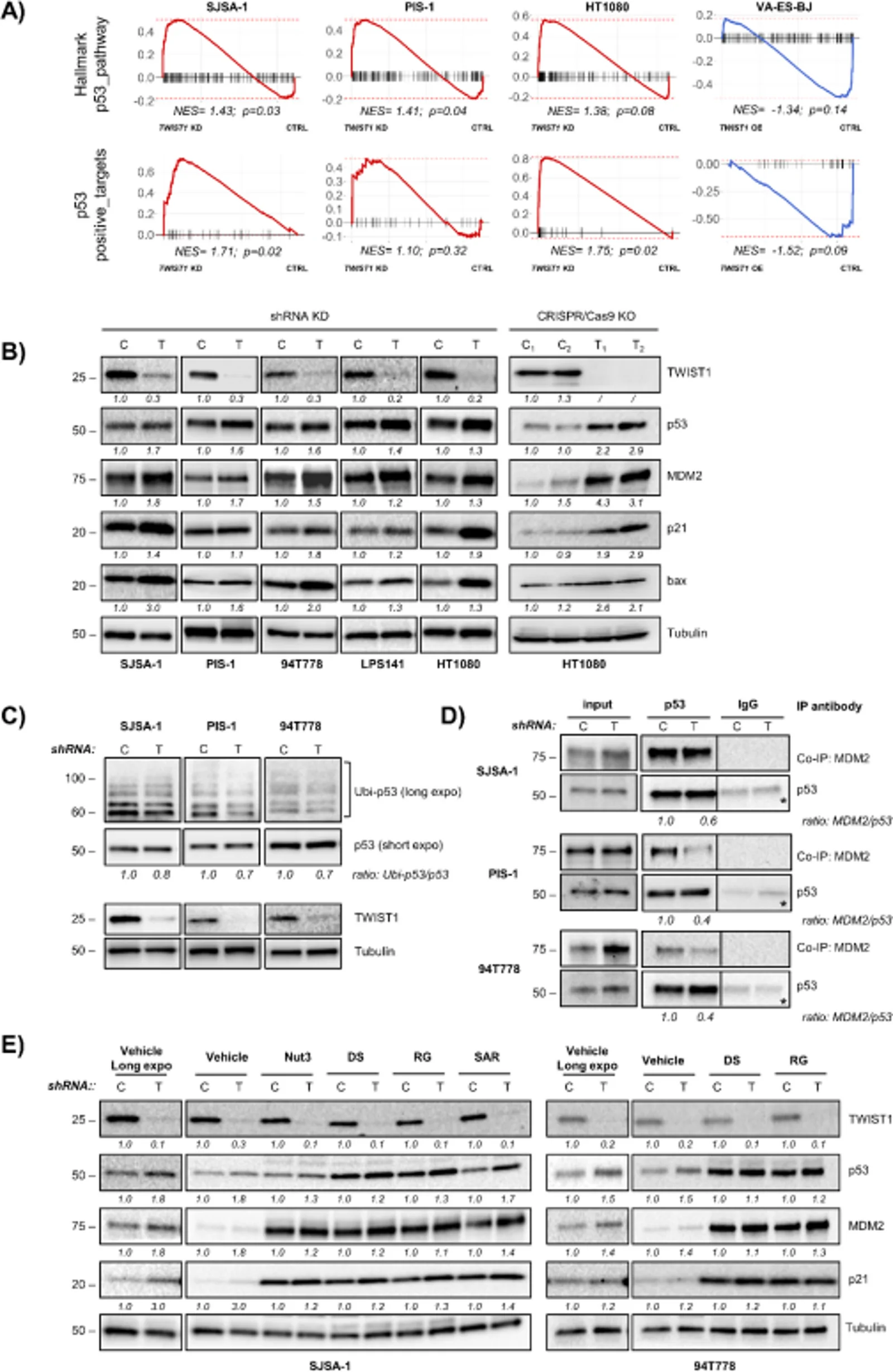
TWIST1 inhibition triggers a p53 response and enhances MDM2i-induced p53 activation. **A** TWIST1 depletion induces hyperactivation of the p53 pathway. Pre-ranked GSEA of genes differentially expressed between *TWIST1*-silenced (*TWIST1* KD) and control (shCTRL) cells revealed a trend toward enrichment of p53 pathway signatures. Conversely, TWIST1 overexpression (TWIST1 OE) in VA-ES-BJ cells tends to attenuate p53 pathway activation. **B** Western blot analysis showing increased protein levels of p53 and downstream targets following TWIST1 inhibition achieved by either shRNA-mediated knockdown (KD) or CRISPR/Cas9 knockout (KO). Two TWIST1 KO clones (T1, T2) and two clones engineered with control, non-targeting guides (C1, C2) are shown. Values below each blot represent the ratio of the integrated densitometric signal of the target protein to that of the corresponding loading control (Tubulin) and expressed relative to the control (**C**) sample, which was set to 1. **C** TWIST1 depletion affects p53 protein ubiquitination. The ratio between the densitometric volume of the p53-associated laddering observed at extended exposure (Ubi-p53, indicated by the square bracket) and that of the main p53 band at short exposure (p53, short expo) is reported. **D** TWIST1 depletion decreases p53:MDM2 interaction. Cells were treated with proteasome inhibitor (10 µM MG132 for 8 h), immunoprecipitated for p53, and probed with the indicated antibodies. The amount of MDM2 bound to p53 (ratio: MDM2/p53) in TWIST1-proficient and -deficient cells is indicated. Asterisks in the control immunoprecipitation (IgG) mark cross-reactivity with the IgG heavy chain. **E** TWIST1 depletion amplifies MDM2i-mediated p53 activation. *TWIST1*-silenced cells were treated with the indicated MDM2i for 4 h and analyzed by immunoblotting for p53 and downstream targets. Drug dosages were as in Fig. 2A. For comparison, a longer exposure of vehicle-treated cells is shown in the left panel. All results were confirmed in at least three independent biological replicates

To gain mechanistic insights into TWIST1 interference with MDM2i activity, we first assessed the impact of TWIST1 on the p53:MDM2 axis, in both the presence and absence of *MDM2* amplification. To this end, we performed pre-ranked GSEA on genes differentially expressed between TWIST1-deficient and TWIST1-proficient cells, using the MSigDB Hallmark p53 pathway gene set [37] and a validated p53-positive target signature [38]. Both analyses revealed a trend toward enrichment of p53 signaling upon TWIST1 knockdown. Conversely, ectopic expression of *TWIST1* in TWIST1-negative VA-ES-BJ cells resulted in a trend toward p53 pathway inhibition (Fig. 3A, Supplementary Table ST2C-D).

Consistent with these transcriptional changes, *TWIST1* knockdown resulted in elevated p53 protein levels and increased expression of p53 target genes (Fig. 3B). RNA-sequencing and RT-qPCR analyses (data not shown) demonstrated that the increase in p53 protein levels detected after TWIST1 inhibition was not attributable to enhanced *TP53* gene transcription, supporting a post-transcriptional mechanism. Although we cannot exclude the possibility that TWIST1 influences *TP53* mRNA translation, previous cycloheximide-chase experiments showed that TWIST1 depletion is associated with an increased p53 half-life [26], consistent with a role for TWIST1 in regulating p53 protein turnover. Extended exposure of p53 immunoblots revealed a ladder-like pattern that was less pronounced in TWIST1-deficient than in TWIST1-proficient cell models (Fig. 3C). This laddering pattern was markedly attenuated following treatment with TAK-243, a selective inhibitor of the ubiquitin-activating enzyme UBA1 (Supplementary Figure S4), indicating that it reflects, at least in part, ubiquitinated forms of p53 [49]. Intriguingly, TWIST1 downregulation was associated with a significant decrease in the amount of MDM2 bound to p53 (Fig. 3C–D; *p* < 0.01). Collectively, these findings are consistent with the hypothesis that TWIST1 may affect p53 signaling by promoting p53 ubiquitination via enhanced p53:MDM2 interaction.

Notably, TWIST1 retained the ability to impinge upon p53 signaling even in the context of MDM2 targeting. In fact, the induction of p53 e its downstream targets by MDM2i was overall more pronounced in TWIST1-depleted cells than in control cells (Fig. 3E). These findings indicate that combined inhibition of TWIST1 and MDM2 further enhances p53 pathway activation.

### TWIST binds MDM2 and forms a trimeric complex with p53

**Fig. 4:**
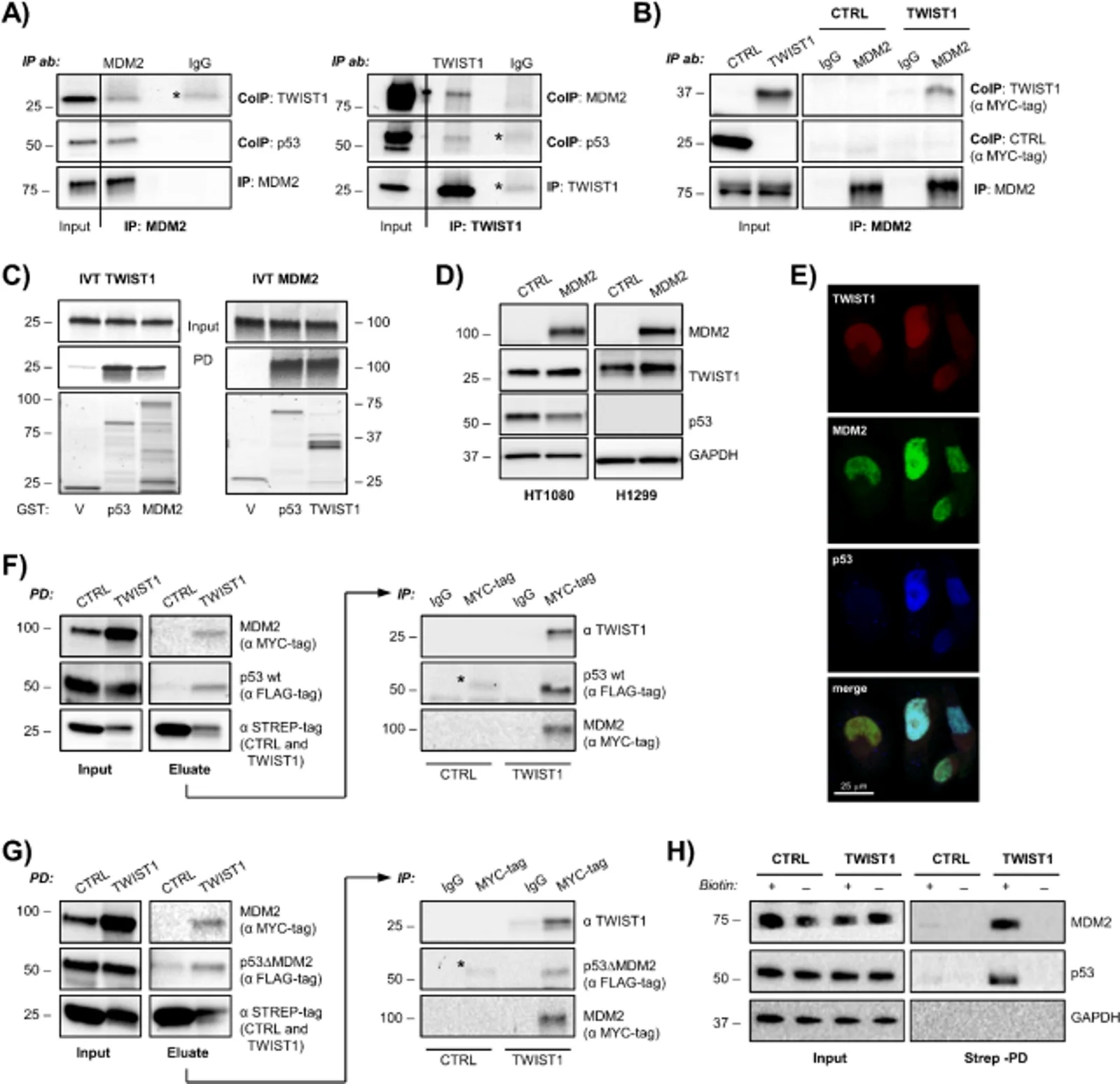
TWIST1 binds MDM2 and forms a trimeric complex with p53 and MDM2. **A** Endogenous TWIST1 and MDM2 co-immunoprecipitate. Nuclear extracts from *MDM2*-amplified SJSA-1 cells were immunoprecipitated with anti-MDM2 (left panel) or anti-TWIST1 (right panel) antibodies and analyzed by western blot. Input lysates are shown. Note: The left and right panels were generated from adjacent regions of the same blot, with the IgG1 control lane shared between both panels and displayed at different exposures. **B** Ectopically expressed TWIST1 co-precipitates with MDM2 in p53-null cells. H1299 cells were co-transfected with MDM2 and either MYC-tagged TWIST1 or MYC-tagged GFP (CTRL), followed by immunoprecipitation with anti-MDM2 or IgG control sera. Immunoblotting antibodies are indicated in brackets. **C** Direct interaction between purified TWIST1 and MDM2 proteins. GST pull down (PD) assays with in vitro translated (IVT) TWIST1 (left) or MDM2 (right) and recombinant GST proteins (p53, MDM2, or TWIST1). GST alone (V) served as a negative control. **D** Ectopic expression of MDM2 does not reduce TWIST1 protein levels in p53-proficient HT1080 or p53-deficient H1299 cells. GAPDH was used as a loading control. **E** Immunofluorescence demonstrates nuclear localization of STREP-tagged TWIST1, MYC-tagged MDM2, and FLAG-tagged p53 in H1299 cells. **F** Serial co-precipitations support the hypothesis of a trimeric p53:TWIST1:MDM2 complex. Cell lysates of H1299 expressing FLAG-p53, MYC-MDM2 together with STREP-TWIST1 or STREP-GFP (CTRL) were affinity-purified with STREP-Tactin sepharose. Immunoblot of the eluate shows co-purification of p53 and MDM2 with TWIST1 (left). The eluate was further immunoprecipitated using anti-MYC-tag antibodies to pull-down MYC-MDM2 and interacting proteins. Both p53 and TWIST1 co-precipitated with MDM2 (right), indicating simultaneous binding. **G** TWIST1 binds MDM2 even when the p53:MDM2 interaction is impaired. The experiment in F was repeated in H1299 cells expressing a FLAG-tagged p53 mutant allele defective for MDM2-binding (p53ΔMDM2). **H** Proximity-dependent biotinylation assay shows that TWIST1-TbID biotinylates p53 and MDM2, supporting direct interactions with both proteins in living cells. Asterisks mark cross-reactivity with antibody heavy and light chains. All results were confirmed in at least three independent biological replicates

By delving deeper into TWIST1:MDM2 interplay we obtained data suggesting a direct physical interaction. Specifically, by immunoprecipitating endogenous MDM2 in SJSA-1 cells, we observed co-precipitation of TWIST1 along with p53 (Fig. 4A, left panel). The apparent TWIST1:MDM2 binding was weaker than p53:MDM2 interaction but reproducible and was observed also when immunoprecipitating for TWIST1 (Fig. 4A, right panel). Importantly, ectopically expressed TWIST1 and MDM2 interacted also in a p53-null cell context (H1299 cells) (Fig. 4B), suggesting that TWIST1:MDM2 binding was not mediated by p53. This hypothesis was validated by GST pull-down experiments using recombinant proteins: in vitro translated (IVT) TWIST1 was pulled down by GST-MDM2; conversely, IVT-MDM2 was captured by GST-TWIST1 (Fig. 4C). Based on these results we concluded that TWIST1 and MDM2 establish a direct physical interaction.

Since TWIST1 is known to be post-transcriptionally regulated through ubiquitin-mediated proteasomal degradation [49], we next assessed whether its interaction with MDM2 could promote TWIST1 degradation. However, ectopic expression of MDM2 did not reduce TWIST1 protein levels (Fig. 4D), nor did it affect TWIST1 mRNA expression (not shown), thereby ruling out a role for MDM2 in mediating TWIST1 turnover.

Having collected evidence that TWIST1 binds both p53 and MDM2, we asked whether TWIST1 binds MDM2 and p53 simultaneously. To tackle this hypothesis, we performed serial co-precipitation experiments. p53-null H1299 cells were engineered to express STREP-tagged TWIST1 together with p53 (FLAG-tagged), and MDM2 (MYC-tagged). Immunofluorescence analysis indicated that all constructs showed nuclear localization (Fig. 4E). STREP pull-down resulted in co-precipitation of p53 and MDM2, corroborating the binding of TWIST1 to both proteins. The resulting eluate was then subjected to a second round of co-precipitations, in this case for MDM2 (using anti-MYC tag antibodies). MDM2 pull-down revealed both p53 and TWIST1 as interacting proteins (Fig. 4F). The binding of TWIST1 to p53 and MDM2 was also detected when a p53ΔMDM2 mutant allele (L22Q/W23S), which is defective for MDM2 binding [44], was used in serial-coprecipitations (Fig. 4G).

Finally, proximity-dependent biotinylation assay (TurboID) [46] was performed to ascertain that TWIST1 direct interaction with MDM2 actually occurs in living cells. To this end, *TWIST1* and CTRL (*GFP*) cDNAs were fused to a FLAG-tagged TurboID biotin ligase. The resulting fusion constructs, *TWIST1*-TbID and CTRL-TbID, were transfected into HEK293T cells, and interacting proteins were recovered by STREP-Tactin bead pull-down. This assay showed that both endogenous MDM2 and p53 were biotinylated by TWIST1-TbID, indicating that TWIST1 physically establishes close connections with both proteins in living cells (Fig. 4H, Supplementary Figure S5). Collectively, these results demonstrate that TWIST1 bridges p53 and MDM2 to form a trimeric nuclear complex.

### The WR domain of TWIST1 is key for MDM2 binding and modulation of MDM2i activity

**Fig. 5:**
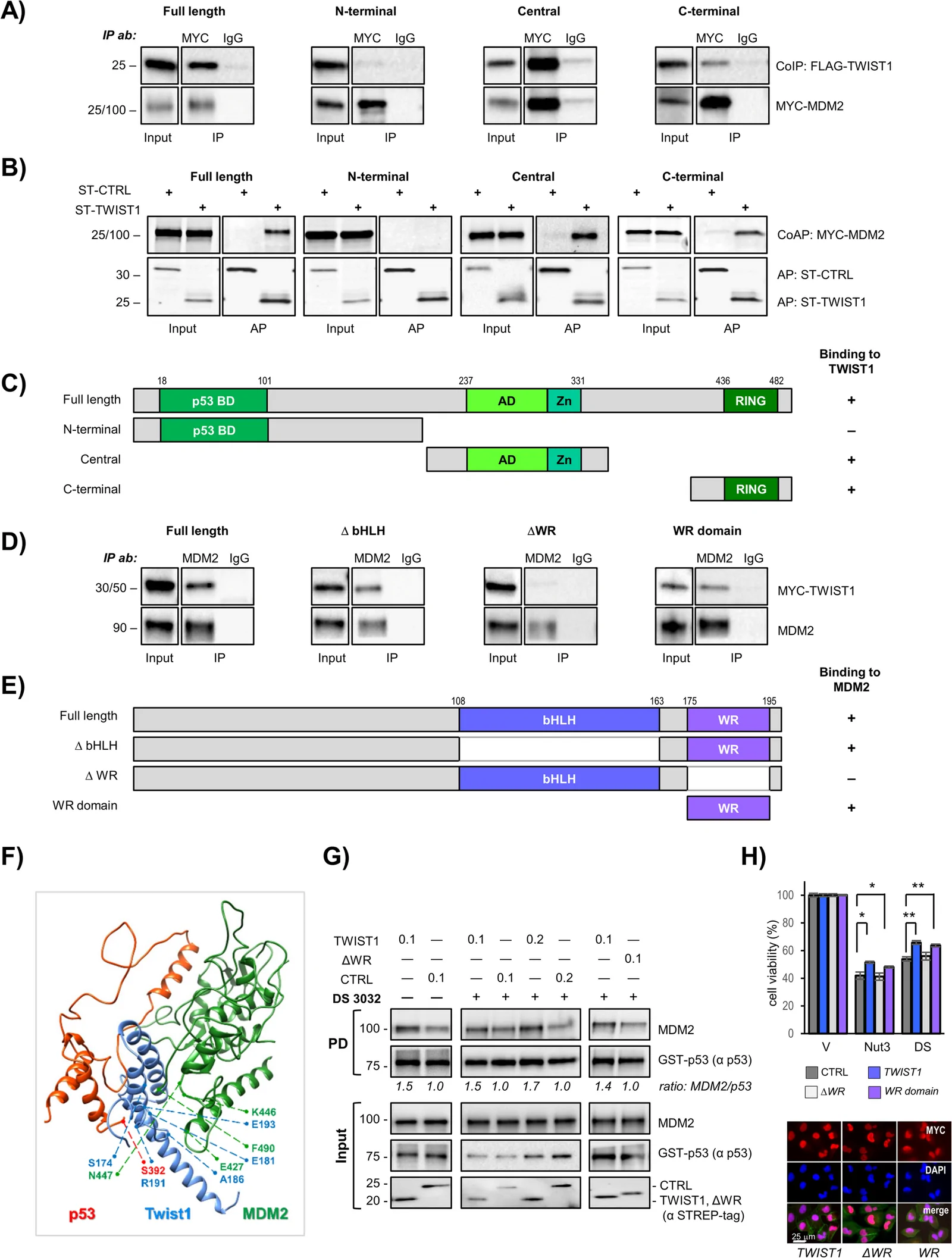
Mapping of TWIST1:MDM2 interaction regions. **A** p53-deficient cells were transfected with FLAG-tagged TWIST1 and MYC-tagged MDM2 (full-length and deletion mutants). Cell lysates were immunoprecipitated with anti-MYC or control IgG antibodies and analyzed by western blot using anti-MYC (for MDM2) or anti-FLAG (for TWIST1) antibodies. Input lysates are shown on the left. **B** Lysates from p53-deficient HCT116 cells expressing MYC-MDM2 deletion mutants were incubated with recombinant STREP-tagged TWIST1 (ST-TWIST1) or GFP (ST-CTRL). STREP-Tactin sepharose was used to purify MDM2 regions interacting with TWIST1. **C** Schematic representation of MDM2 and relative deletion mutants used to map TWIST1-binding domains. Effective binding is indicated on the right. Abbreviations: p53 BD, p53-binding domain; AD, acidic domain; Zn, zinc finger; RING, RING finger domain. **D** HEK293T cells were transfected with MDM2 and MYC-tagged TWIST1 (full-length and derived mutants). Lysates were immunoprecipitated with anti-MDM2 or control antibodies (IgG) and analyzed by western blot. **E** Schematic representation of TWIST1 and relative mutants used to map MDM2-binding domains. Abbreviations: bHLH, basic helix-loop-helix domain; WR, WR domain. **F** Structural docking model of the ternary complex formed by TWIST1 (blue), p53 (orange), and MDM2 (green). To facilitate visual inspection, the image focuses on TWIST1-mediated interactions, showing representative contacts between the TWIST1 WR domain and the C-terminal regions of p53 and MDM2 (**G**) GST pull-down assay showing that recombinant TWIST1 enhances the interaction between MDM2 and p53, both in the absence and presence of DS-3032. STREP-MDM2 was pre-incubated with DS-3032 (+) or vehicle (-) for 2 h, followed by addition of GST-p53 together with STREP-tagged TWIST1 (ST-TWIST1) or RFP used as control protein (ST-CTRL). Complexes were isolated by GST pull-down and analyzed by immunoblot. Recombinant TWIST1 increases MDM2 binding to p53. Two different TWIST1 doses (0.1 and 0.2 µg) are shown. The right panel shows that deletion of the WR domain impairs the ability of TWIST1 to enhance the p53:MDM2 interaction (MYC/STREP-tagged TWIST1ΔWR). Results were reproducible across at least three independent experiments. **H** The WR domain of TWIST1 is necessary and sufficient to impair MDM2i efficacy. VA-ES-BJ cells were engineered to express MYC-tagged TWIST1, full-length and mutants (ΔWR and WR). Cells were then treated with nutlin-3a (10 µM) or DS-3032 (0.1 µM) for 48 h and cell viability was determined by SRB assay (upper panel; see also Supplementary Figure S6). Nuclear localization (lower panel) was verified by anti-MYC immunofluorescence (red), with nuclei stained using DAPI (blue) and cytoskeleton stained with FITC-phalloidin (green). All results were confirmed in at least three independent biological replicates

To map MDM2 domains involved in the interaction with TWIST1, we performed co-immunoprecipitation (Fig. 5A) and STREP-Tactin pull-down assays (Fig. 5B) using MYC-tagged MDM2 deletion mutants. These analyses indicated that MDM2 establishes contacts with TWIST1 through a region spanning the acidic and C-terminal RING finger domains (Fig. 5C).

Reciprocally, TWIST1 engaged the C-terminal WR domain to interact with MDM2. In fact, a small peptide corresponding to the TWIST1 WR domain, displayed from the active site loop of thioredoxin, was sufficient to bind MDM2 (Fig. 5D-E). Interestingly, TWIST1 C-terminus is also involved in the binding to p53 [26], and molecular docking simulations corroborated the notion that this TWIST1 domain makes contacts with the C-terminal region of both proteins, thus supporting the trimeric complex model (Fig. 5F). To validate the biological relevance of TWIST1:MDM2 connection, we performed in vitro binding assays using recombinant proteins (Fig. 5G). In the presence of recombinant TWIST1, the amount of MDM2 bound to p53 was increased, both in the absence and presence of DS-3032. No significant increase was observed with the WR domain deletion mutant (TWIST1ΔWR), corroborating the relevance of TWIST1 interaction with p53 and MDM2 (Fig. 5G). Accordingly, while ectopic expression of TWIST1 in VA-ES-BJ cells impaired MDM2i efficacy, the TWIST1ΔWR mutant failed to do so. Conversely, the TWIST1 WR peptide recapitulated the effect of full-length TWIST1 (Fig. 5H, Supplementary Figure S6).

**Fig. 6:**
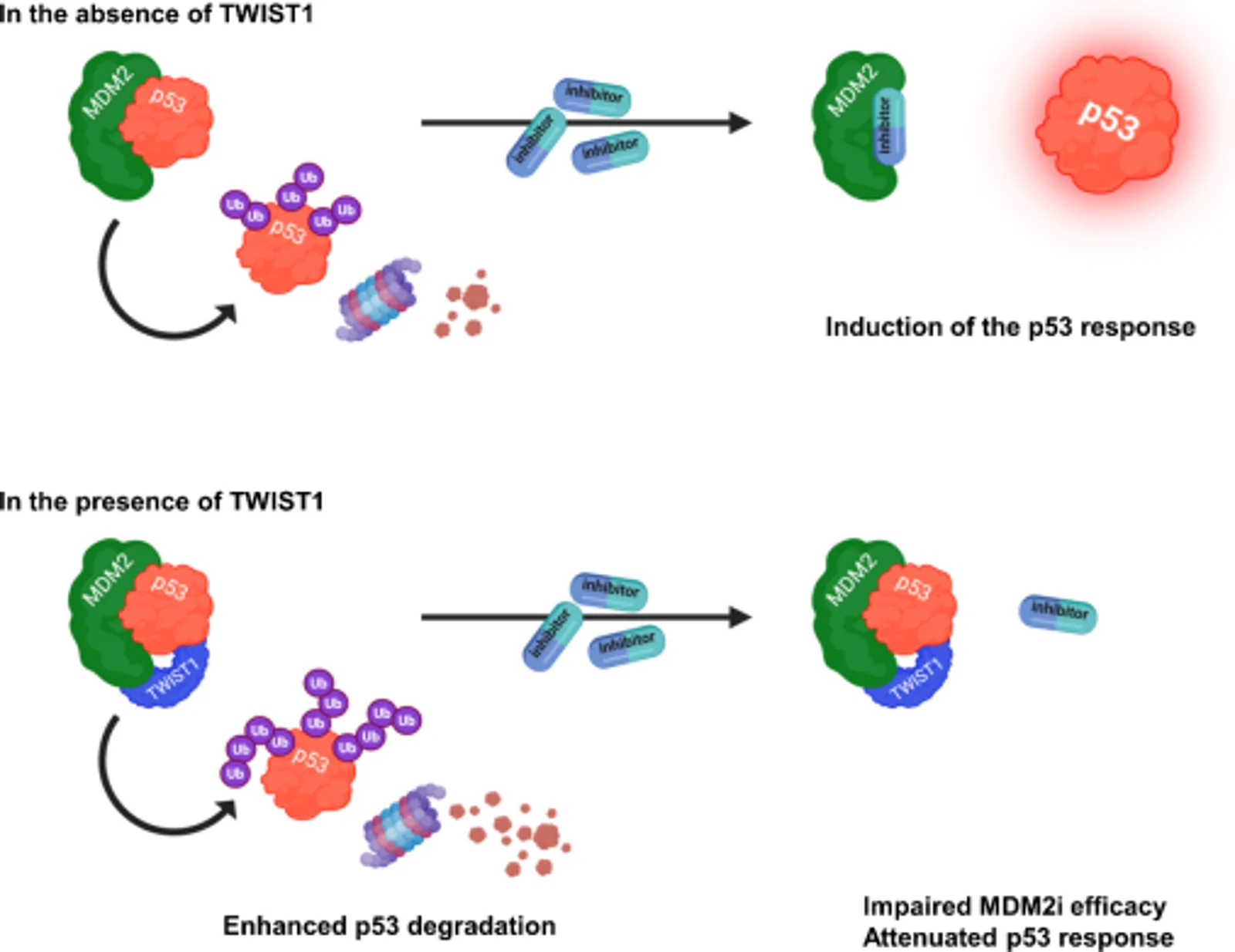
Schematic representation of the proposed role of TWIST1 in the response to MDM2i. In the absence of TWIST1, MDM2 binds p53 and targets it to proteasomal degradation. MDM2i hinder the p53:MDM2 interaction, leading to p53 stabilization and activation of the p53 response. TWIST1, by bridging p53 and MDM2, promotes MDM2-mediated degradation of p53, thereby hampering the efficacy of MDM2i. The figure was created with BioRender.com

Overall, our results support a model in which TWIST1 interplay with the p53:MDM2 axis modulates the response to MDM2i (Fig. 6) and suggest TWIST1 suppression as a potential strategy to enhance the therapeutic efficacy of these inhibitors.

## Discussion

MDM2 is a central oncogenic driver in *TP53* wild-type/*MDM2*-amplified tumors and is the focus of intense investigation as a therapeutic target [18, 20]. Several inhibitors designed to disrupt the p53:MDM2 interaction have entered clinical trials and new modalities aimed at promoting MDM2 degradation, such as PROTACs, are under active development [50–53]. Given the strong dependency on MDM2 to dampen the p53 response, *TP53* wild-type/*MDM2*-amplified sarcomas were considered ideal candidates for MDM2i treatments. Unfortunately, despite a solid rationale, these inhibitors have thus far failed to demonstrate the anticipated therapeutic benefits in clinical trials [13, 19]. In particular, the MDM2 inhibitors that reached phase III trials in sarcomas (milademetan and brigimadlin) failed to meet the primary endpoint of improved progression-free survival compared with the standard of care, although meaningful signals of activity were observed in some patients. In fact, the investigators involved in the Brightline-1 trial (NCT05218499, phase II/III, randomized, open-label, multicenter study comparing brigimadlin to doxorubicin in advanced dedifferentiated liposarcoma) observed that response endpoint was not formally met due to hierarchical ranking of endpoints, and indeed the objective response and disease control rates were numerically superior with brigimadlin compared to doxorubicin [14, 17]. Similarly, higher response rates were observed in the milademetan arm of the phase III MANTRA trial in dedifferentiated liposarcoma, although progression-free survival was not statistically different compared with the trabectedin control arm [14, 16, 54].

Several factors may have contributed to the failure to meet the primary endpoint in these studies. Beyond complex patient variables, the limited characterization of dose- and schedule-dependent efficacy, particularly with respect to the p53:MDM2 feedback loop, likely played a key role [14, 54]. Definitively, the sole condition of being *TP53* wild-type/*MDM2*-amplified has proven inadequate as a predictor of therapeutic response. These findings underscore the urgent need for a deeper understanding of the biological determinants that influence the clinical activity of MDM2i, to enable rational patient selection and stratification based on individual sensitivity, and to guide the design of combination therapies [14, 55].

In this work, we provide evidence that TWIST1 is one of the determinants that affect MDM2i efficacy. We have previously shown that TWIST1 contributes to the functional inactivation of p53 in *TP53* wild-type sarcomas. Specifically, we have demonstrated that TWIST1 interacts with p53 and, by preventing key phosphorylations, facilitates p53 turnover [26]. Here, we demonstrate that *TWIST1* gene expression levels and signaling activity inversely correlate with sensitivity to MDM2i in *TP53* wild-type sarcoma cell models. Inhibition of TWIST1 expression, by either shRNA-mediated knockdown or CRISPR/Cas9 knockout, enhances cell responsiveness to different MDM2i both in vitro and *in vivo.* This is paralleled by an over-induction of the p53 pathway. Reciprocally, TWIST1 overexpression impairs MDM2i efficacy. These findings support a role for elevated TWIST1 expression in intrinsic resistance to MDM2i and point to TWIST1 as a potential negative predictive biomarker of MDM2i activity.

Intriguingly, a recent study proposed *TWIST1* amplification as a positive predictive marker of MDM2i activity in intimal sarcomas [56]. In this work, the authors reported a partial response to milademetan in 2 out of 10 intimal sarcoma patients, both exhibiting increased *TWIST1* gene copy number. Surprisingly, *TWIST1* mRNA expression did not correlate with treatment response. Here, we provide compelling evidence that TWIST1 negatively impacts MDM2i efficacy in the context of sarcomas, including intimal sarcoma cells (PIS-1 cell line). Notably, the amplified chromosomal region encompassing the *TWIST1* locus also harbors several poorly characterized non-coding RNAs and distal regulatory elements. It is possible that the amplification of these elements -rather than TWIST1 itself- underlies the observed sensitivity to milademetan.

For a mechanistic standpoint, we show that TWIST1 directly binds both p53 and MDM2, forming a trimeric complex that facilitates the p53:MDM2 interaction and thereby promotes p53 degradation. TWIST1 confers resistance to MDM2 inhibitors through its WR domain, a mechanism that is distinct from its canonical EMT-promoting function, which involves transcriptional regulation of target genes via bHLH domain [57]. Notably, Li et al. [58] reported that depletion of TWIST1 increases the sensitivity of MCF7 breast cancer cells to adriamycin, a phenomenon that is paralleled by reduced binding between p53 and MDM2. Here, we provide experimental evidence that TWIST1 forms a complex with both p53 and MDM2, indicating a direct role in modulating their interaction.

Based on our findings, we propose a model where, by bridging p53 and MDM2 and promoting p53 degradation, TWIST1 attenuates the capacity of MDM2i to prevent p53:MDM2 coupling and to induce the p53 tumor suppressive response. Other proteins have been reported to modulate p53:MDM2 complex formation and activity. For instance, a p53:SNAI2:MDM2 trimeric complex has been shown to form specifically to facilitate MDM2-mediated degradation of SNAI2 [59]. In contrast, we found that the binding to MDM2 does not target TWIST1 for degradation; rather, the trimeric complex promotes the degradation of p53. This mechanism is similar to that described for the p53:YY1:MDM2 complex, in which YY1 enhances MDM2-mediated ubiquitination of p53 [60]. We show that the interaction with p53 and MDM2 is crucial for TWIST1 to hamper MDM2i efficacy. In fact, a TWIST1 deletion mutant that lacks the binding region for p53 and MDM2 fails to attenuate MDM2i activity whilst a peptide encoding this binding region is sufficient to hamper MDM2i efficacy.

Intriguingly, we found that harmine phenocopies TWIST1 knockdown in potentiating MDM2i efficacy. Harmine, a natural alkaloid with diverse biological activities, has been proposed as a TWIST1 inhibitor due to its capacity to induce TWIST1 protein degradation [47, 48]. Harmine is not an approved drug, but preclinical studies indicate neuroprotective, anti-inflammatory, and anti-tumor effects, and clinical trials are underway to assess its pharmacokinetics, pharmacodynamics, and safety [61].

## Conclusions

In summary, our study shows that TWIST1 forms a trimeric complex with p53 and MDM2 and identifies TWIST1 as a critical mediator of primary resistance to MDM2i in *TP53* wild-type sarcomas. Furthermore, we provide evidence that TWIST1 targeting, including harmine-mediated inhibition, significantly enhances sarcoma cell sensitivity to MDM2 blockade. Unfortunately, the lack of access to molecular profiling data from company-sponsored clinical trials of MDM2 inhibitors in sarcomas prevents validation of our findings in human samples, representing a limitation of the study. Nevertheless, our results provide a strong preclinical rationale for evaluating TWIST1 as a potential predictive biomarker and for the future clinical assessment of combined MDM2 inhibitor and TWIST1-targeted therapies in sarcomas.

## Supplementary Information

Supplementary Material 1.

Supplementary Material 2.

Supplementary Material 3.

Supplementary Material 4.

Supplementary Material 5.

## Data Availability

Transcriptome data generated in this study are provided as Supplementary Table ST2. Raw data are available upon request from the corresponding author. For the GDSC sarcoma cell line cohort, pTPM were retrieved from the DepMap Public 24Q4 dataset (https://depmap.org/portal/data_page/?tab=allData&releasename=DepMap%20Public%2024Q4&filename=OmicsExpressionProteinCodingGenesTPMLogp1.csv) and IC50 values for nutlin-3a (GDSC1 dataset) retrieved from the GDSC database (https://www.cancerrxgene.org/compound/Nutlin-3a%20(-)/1047/overview/ic50?screening_set=GDSC1).

## Abbreviations

MDM2i: MDM2 inhibitors
WDLPS: Well-differentiated liposarcoma
DDLPS: Dedifferentiated liposarcoma
IS: Intimal sarcoma
PROTAC: PROteolysis-Targeting Chimera
GDSC: Genomics of drug sensitivity in cancer
IC50: Half maximal inhibitory concentration
pTPM: Protein-coding transcripts per million
DMSO: Dimethyl Sulfoxide
NUT3: Nutlin-3a
DS: DS-3032 (milademetan)
RG: RG7388 (idasanutlin)
SAR: SAR405838 (MI-77301)
BI: BI 907828 (brigimadlin)
SRB: Sulforhodamine B
TbID: TurboID
