# Supplementary material for: TWIST1 bridges p53 and MDM2 and impairs the efficacy of MDM2 inhibitors
Source: J Exp Clin Cancer Res. 2026 Jul 16;45:213. doi: 10.1186/s13046-026-03780-w (PMC13656556; doi:10.1186/s13046-026-03780-w)
Supplement: Supplementary file 1 [file 13046_2026_3780_MOESM5_ESM.pdf]

Supplementary Figures

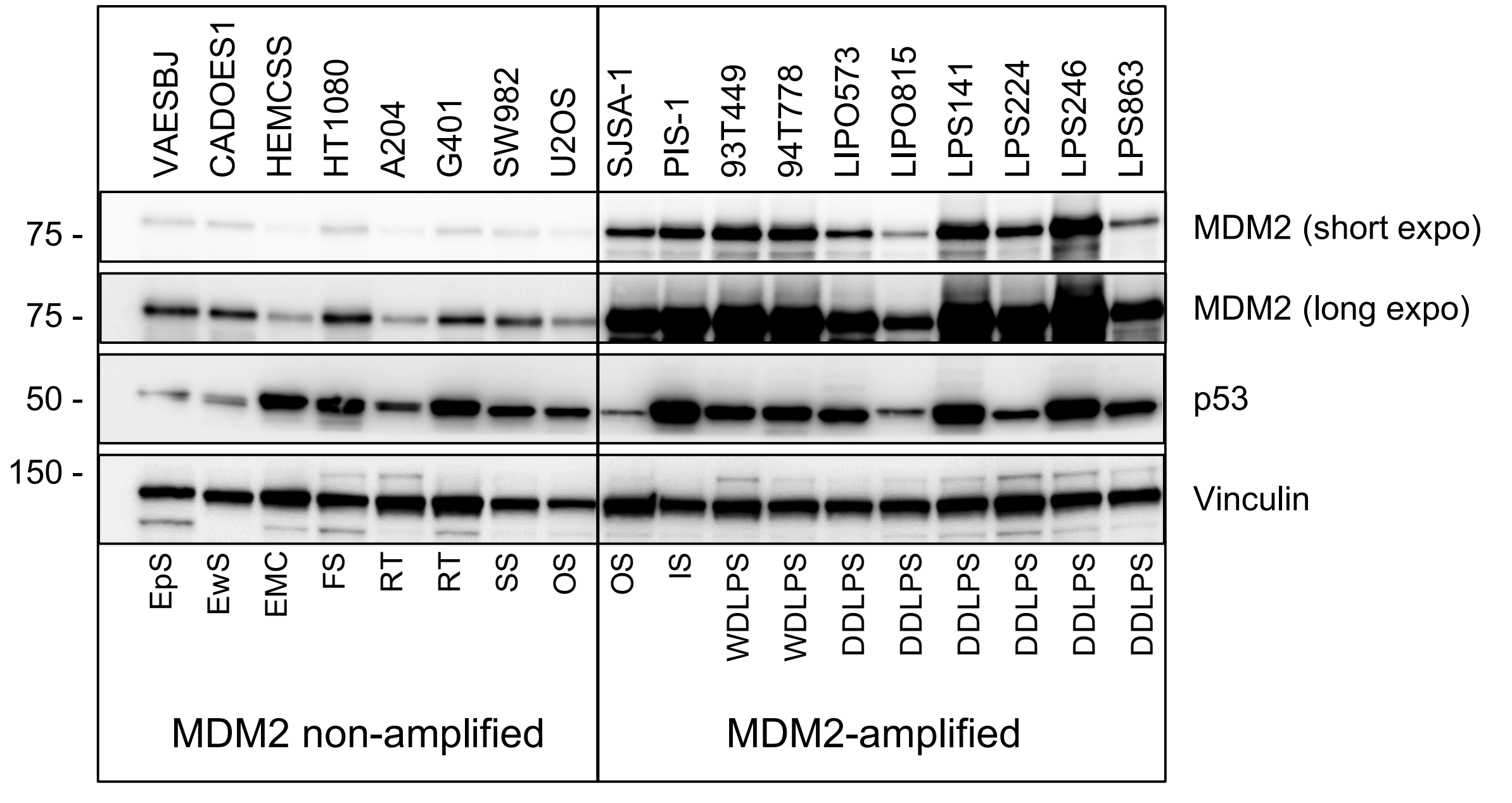

Supplementary Figure S1. Immunoblot analysis of MDM2 and p53 expression in the cell lines shown in Figure 1, excluding KYM1 and HUO9, which were not available in our laboratory. Vinculin was used as a loading control. Two exposures of MDM2 are presented to emphasize the differences in expression between MDM2-non-amplified and MDM2-amplified cell lines. Abbreviations: EpS, Epithelioid Sarcoma; EwS, Ewing Sarcoma; EMC, Extraskeletal Myxoid Chondrosarcoma; FS, Fibrosarcoma; RT, Rhabdoid Tumor; SS, Synovial Sarcoma; OS, Osteosarcoma; IS, Intimal Sarcoma; WDLPS, Well Differentiated Liposarcoma; DDLPS, Dedifferentiated Liposarcoma.

Supplementary Figures

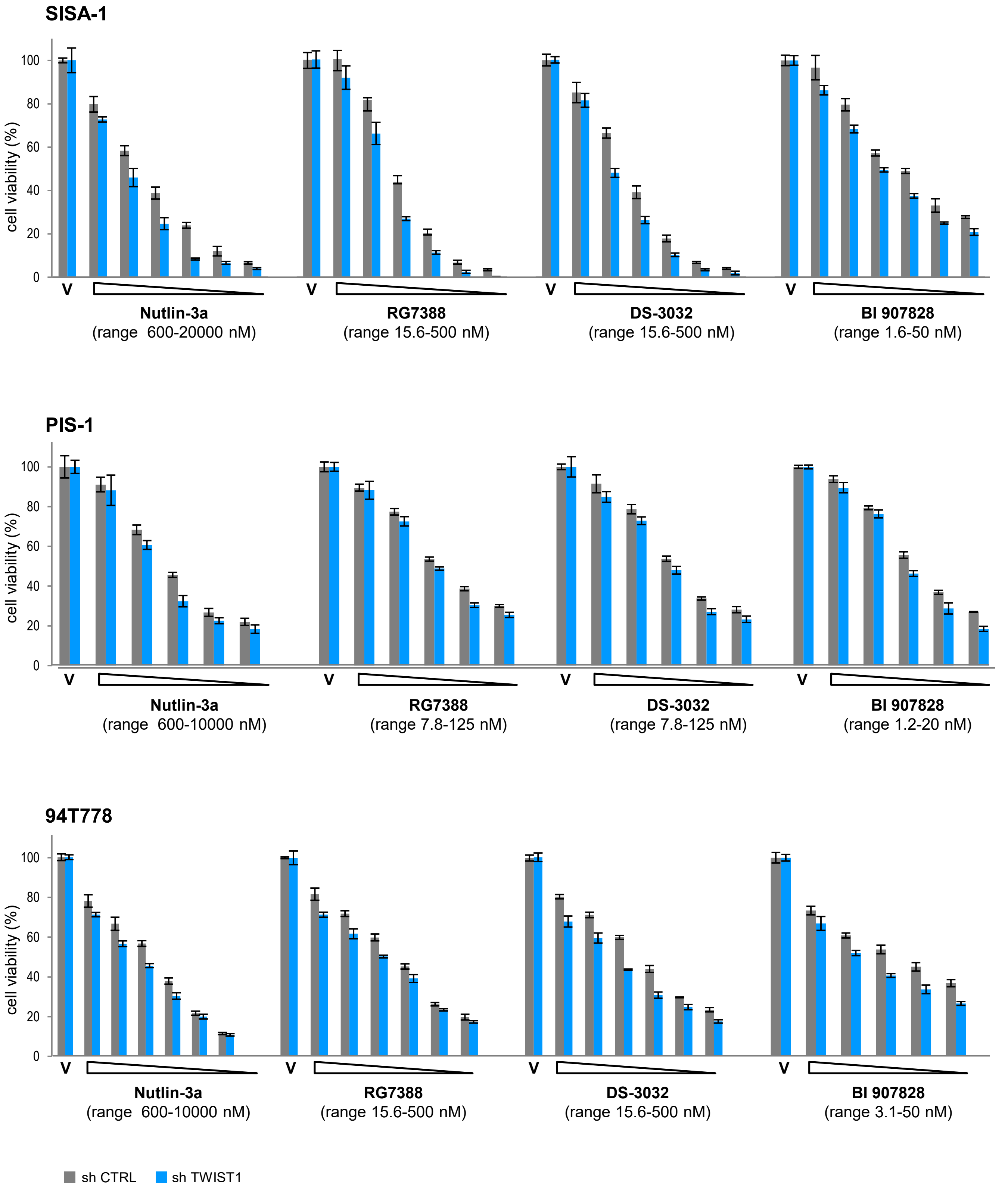

Figure S2. Representative dose–response analyses. Equal numbers of cells expressing either shCTRL (grey) or sh*TWIST1* (blue) were treated with the indicated MDM2i across a two-fold concentration series. Cell viability was measured by SRB assay after 48 hours. *TWIST1*-silenced cells showed a statistically significant ($p < 0.05$) increase in sensitivity to all tested MDM2 inhibitors at all concentrations.

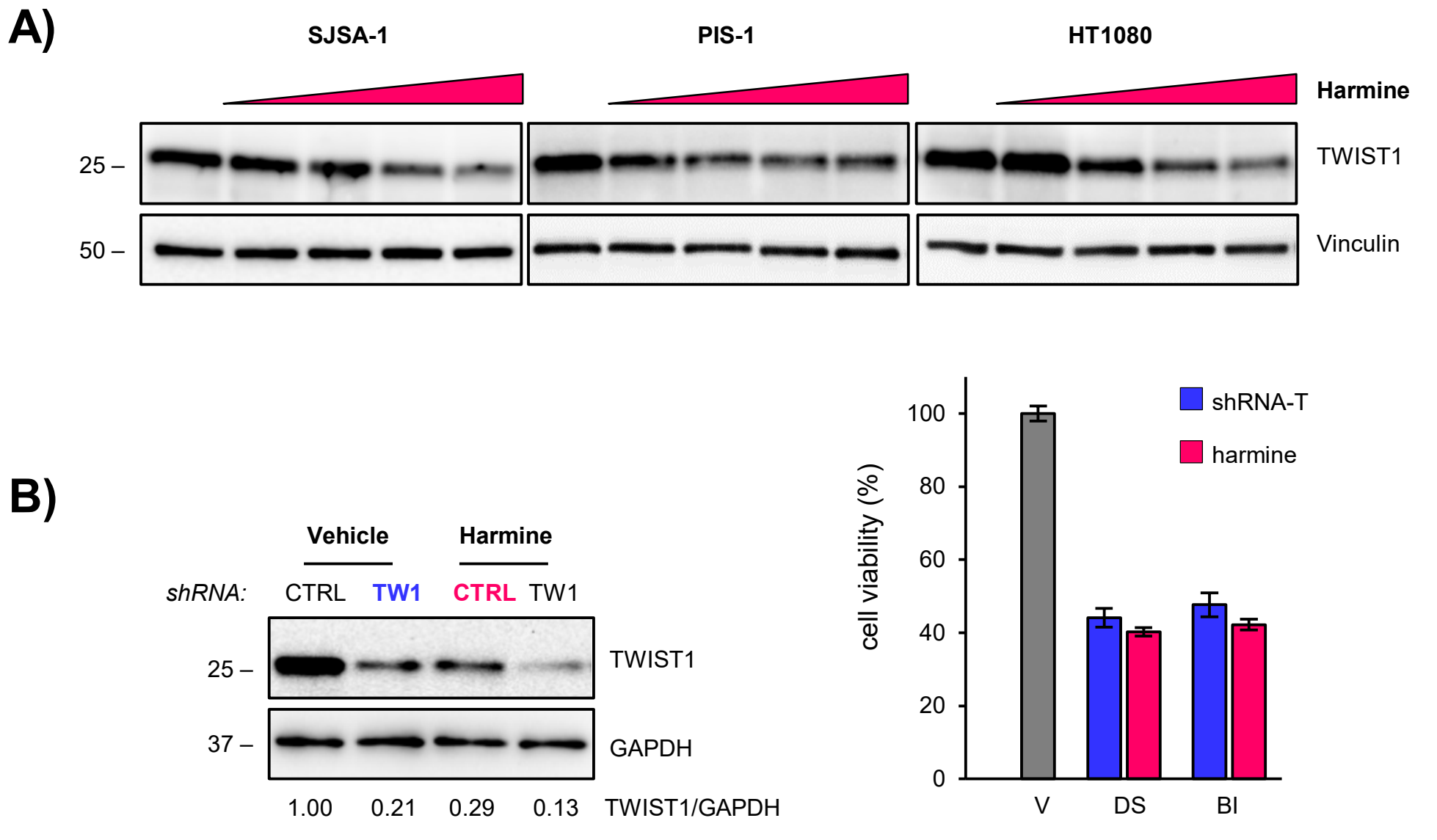

Figure S3. Harmine mimics TWIST1 genetic inhibition in modulating MDM2i sensitivity. (A) Immunoblot analysis of sarcoma cell lines treated for 48 hours with increasing concentrations of harmine (0, 5, 10, 20, and 40 µM) shows a dose-dependent decrease in TWIST1 protein levels. (B) SJSA-1 cells with comparable TWIST1 protein reduction achieved through genetic silencing (vehicle-treated shRNA TW1) or chemical inhibition (harmine-treated shRNA CTRL) (left panel) show similar sensitivity to MDM2i (50 nM DS-3032; 10 nM BI 907828; 48h treatment) (right panel) .

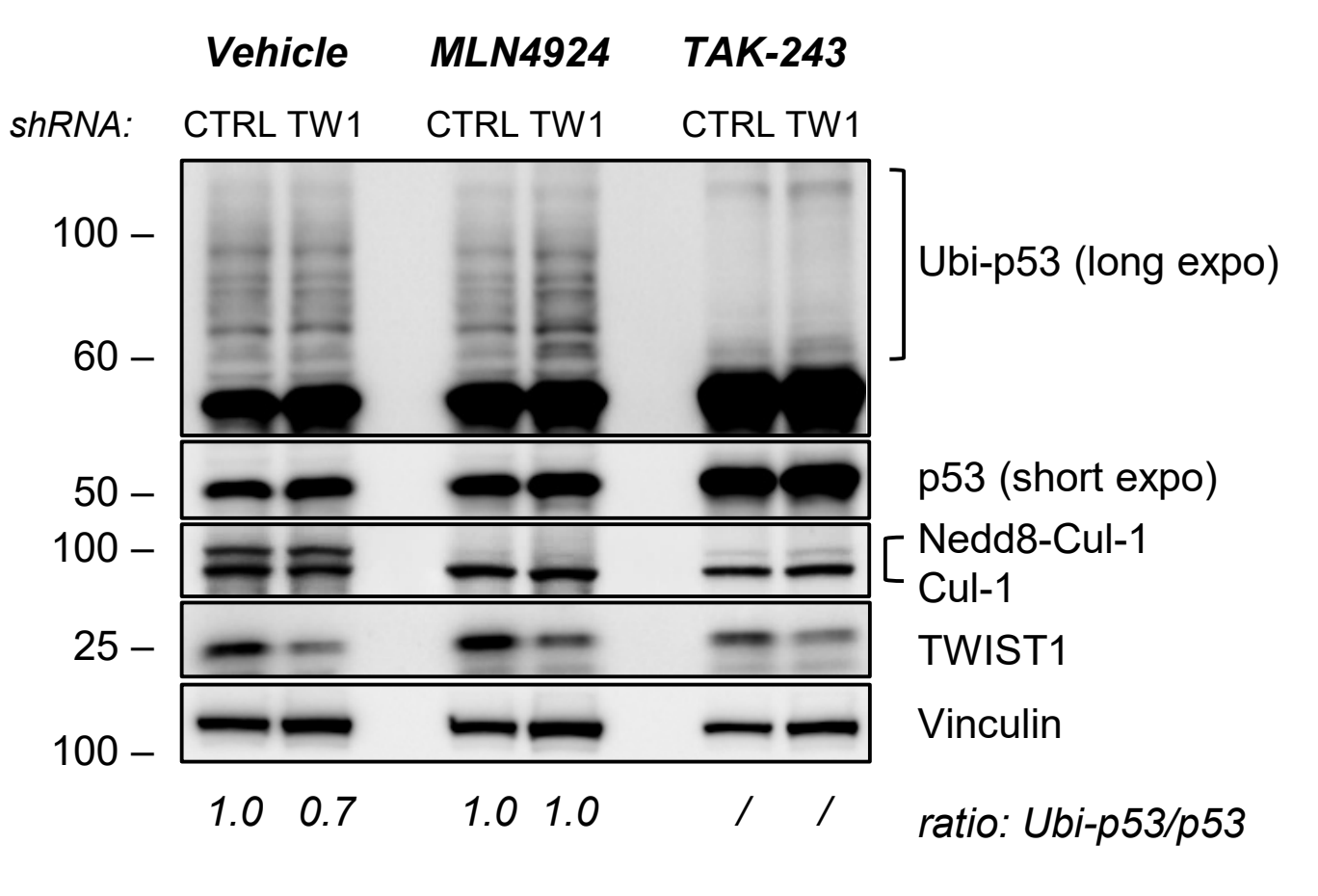

Figure S4. Reduced p53 laddering in *TWIST1*-silenced cells reflects decreased ubiquitinated p53 species. SJSA-1 control (CTRL) and *TWIST1*-silenced cells (TW1) were treated for 3 h with vehicle (DMSO), MLN4924, or TAK-243 (5 µM). Inhibition of neddylation by MLN4924 did not alter p53 laddering in either cell model. In contrast, TAK-243 significantly affected the laddering pattern in both TWIST1-proficient and TWIST1-deficient cells, indicating that most of the higher-molecular-weight p53 bands correspond to ubiquitination-dependent species. The ratio between the densitometric volume of the p53-associated laddering detected at extended exposure (Ubi-p53, indicated by the square bracket) and that of the main p53 band at short exposure (p53, short expo) is reported. Effective inhibition of ubiquitination was confirmed by p53 stabilization following TAK-243 treatment. Conversely, the higher-molecular-weight band corresponding to neddylated CUL1 was completely abolished by MLN-4924, confirming effective inhibition of neddylation. The partial reduction in CUL1 neddylation upon TAK-243 treatment is consistent with previously described crosstalk between ubiquitination and cullin neddylation pathways (Enchev et al. Nat Rev Mol Cell Biol. 2015, 16:30-44).

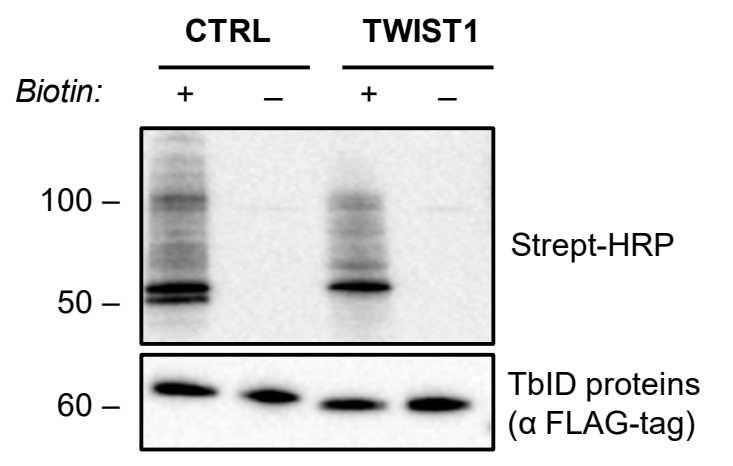

Figure S5. Biotinylation labeling effectiveness by the TurboID constructs used in this work. HEK293T cells were transfected with FLAG-tag TurboID (TbID) plasmids encoding either TWIST1 or control GFP (CTRL). Streptavidin-HRP immunoblotting confirmed strong biotinylation activity from the TbID constructs.

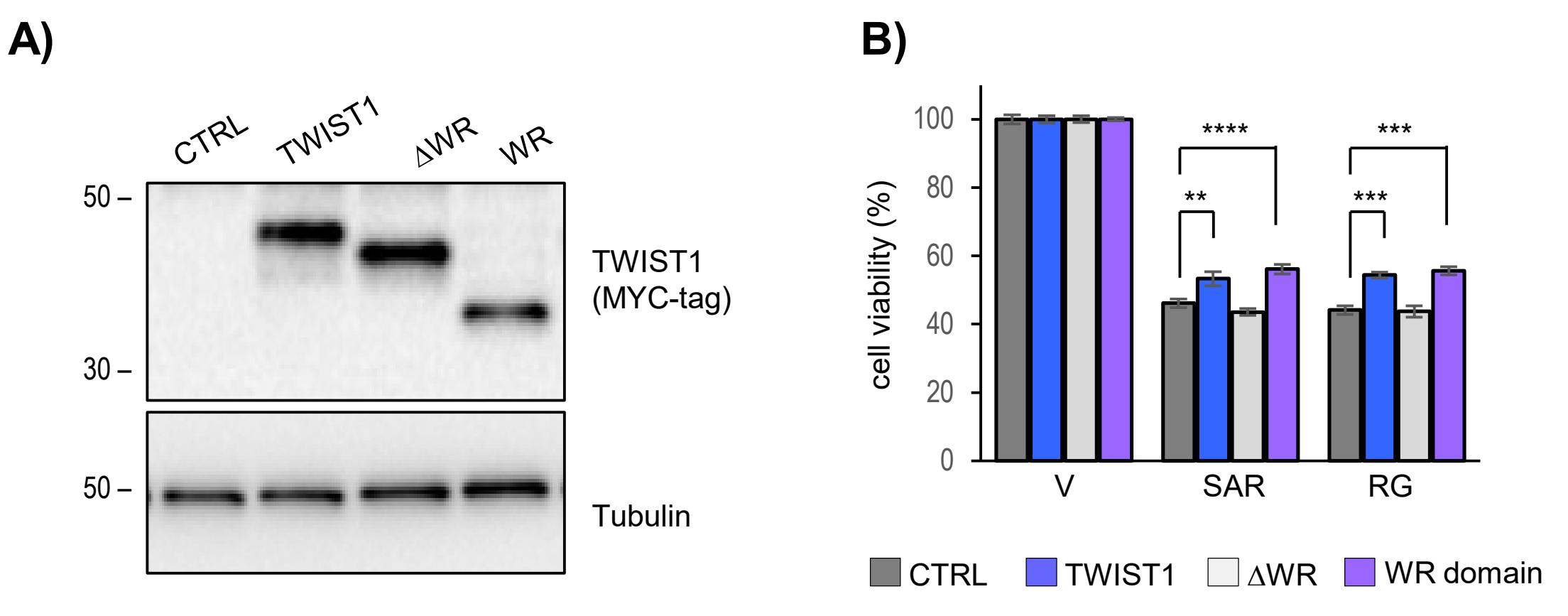

Figure S6. (A) Western blot analysis of VA-ES-BJ cells engineered to ectopically express TWIST1, the WR domain deleted TWIST1 mutant (ΔWR), and the peptide corresponding to the WR domain (see Figure 5H). (B) Sensitivity to SAR405838 (SAR, 0.5 μM, 48h), and RG7388 (RG, 0.5 μM, 48h) of the VA-ES-BJ cell models shown in (A). Statistical significance is indicated as follows: *, $p \leq 0.05$, **, $p \leq 0.01$, ***, $p \leq 0.001$, ****, $p \leq 0.0001$.
